# Correlating Mediterranean shallow water deposits with global Oligocene–Miocene stratigraphy and oceanic events^☆^

**DOI:** 10.1016/j.gloplacha.2013.09.018

**Published:** 2013-12

**Authors:** Markus Reuter, Werner E. Piller, Marco Brandano, Mathias Harzhauser

**Affiliations:** a Institute for Earth Sciences, University of Graz, Heinrichstrasse 26, 8010 Graz, Austria; b Dipartimento di Scienze della Terra, Università di Roma “La Sapienza”, P. Aldo Moro 5, 00185 Roma, Italy; c Natural History Museum Vienna, Geological-Paleontological Department, Burgring 7, 1010 Vienna, Austria

**Keywords:** shallow-marine carbonates, stratigraphic correlation, paleoceanographic events, Oligocene–Miocene, Maiella Platform, Mediterranean Sea

## Abstract

Shallow-marine sediment records have the strong potential to display sensitive environmental changes in sedimentary geometries and skeletal content. However, the time resolution of most neritic carbonate records is not high enough to be compared with climatic events as recorded in the deep-sea sediment archives. In order to resolve the paleoceanographic and paleoclimatic changes during the Oligocene–Miocene transition in the Mediterranean shallow water carbonate systems with the best possible time resolution, we re-evaluated the Decontra section on the Maiella Platform (central Apennines, Italy), which acts as a reference for the correlation of Oligocene–Miocene shallow water deposits in the Mediterranean region. The 120-m-thick late Oligocene–late Miocene carbonate succession is composed of larger foraminiferal, bryozoan and corallinacean limestones interlayered with distinct planktonic foraminiferal carbonates representing a mostly outer neritic setting. Integrated multi-proxy and facies analyses indicate that CaCO_3_ and total organic carbon contents as well as gamma-ray display only local to regional processes on the carbonate platform and are not suited for stratigraphic correlation on a wider scale. In contrast, new biostratigraphic data correlate the Decontra stable carbon isotope record to the global deep-sea carbon isotope record. This links relative sea level fluctuations, which are reflected by facies and magnetic susceptibility changes, to third-order eustatic cycles. The new integrated bio-, chemo-, and sequence stratigraphic framework enables a more precise timing of environmental changes within the studied time interval and identifies Decontra as an important locality for correlating not only shallow and deep water sediments of the Mediterranean region but also on a global scale.

## Highlights

• We establish a new age model for the Oligo-Miocene Decontra section.
• The local carbon isotope record is correlated with the global δ^13^C curve.
• This correlates Mediterranean shallow water records with oceanic/climatic events.

## 1 Introduction

For stratigraphy of the Cenozoic Era, the Mediterranean is of peculiar meaning because nearly all stages are defined with their GSSPs in this region. These GSSPs were established in deep water successions, many of them in Italy, which have a complete record and planktonic fossils afford the necessary biostratigraphic resolution (Hilgen et al., 2012; Vandenberghe et al., 2012). More recently, this time resolution was distinctly enhanced by astrochronology, which allows to resolve at a scale of thousands of years (e.g., Shackleton et al., 2000; Hilgen et al., 2003; Iaccarino et al., 2004, 2011; Hilgen, 2008; Turco et al., 2011a). Due to this high time resolution and more stable environmental conditions paleoceanographic and paleoclimatic interpretations for the Cenozoic mainly rest on isotope studies of deep water deposits (e.g., Miller et al., 1991; Zachos et al., 2001; Billups and Schrag, 2003; Holbourn et al., 2007). In contrast to deep water successions, there is an intrinsic limitation regarding achievable age resolution of shallow water deposits (Mutti et al., 2010). Hence, Cenozoic shallow-marine sedimentary successions in the Mediterranean region are predominantly lithostratigraphically classified. Accurate chronostratigraphic correlations are generally impeded by the stratigraphically incomplete record and the less precise biostratigraphic resolution in shallow and marginal marine settings (Mutti et al., 2010). Accordingly, the stratigraphic distribution of specific heterozoan and chlorozoan carbonate facies associations in the central Mediterranean and their relationship to the Neogene climate trends and particular paleoceanographic conditions in the Mediterranean Sea mainly rely on δ^13^C records (Jacobs et al., 1996; Mutti et al., 1997, 1999, 2006; John et al., 2003; Kocsis et al., 2008; Brandano et al., 2010). However, a comparison between sections evidences specific regional offsets and, generally, an offset with the global carbon isotope record (Mutti et al., 1997, 1999; Brandano et al., 2010). Two main factors may explain these discrepancies between the Mediterranean shallow-marine and the global deep-sea isotope curves: (1) an inadequate or low resolution stratigraphy; or (2) the specific paleogeographic and paleoceanographic changes, which occur in this region during the Oligocene–Miocene (Pedley, 1987; Brandano and Corda, 2002; Bosellini and Perrin, 2008; Brandano et al., 2009a,b, 2010).

The Maiella (Abruzzi, central Apennines, Italy) represents a tectonically relative stable carbonate platform in the central Mediterranean Sea (Vecsei and Sanders, 1997). Due to its isolated position (Mutti et al., 1999; Brandano et al., 2012) and long-term stratigraphic record of shallow water sediments (Vecsei and Sanders, 1997) this carbonate platform is well suited to identify local processes possibly overprinting regional or global signals in neritic carbonates. The Oligocene–Miocene Decontra section, at the northwestern platform margin, is a reference section for the correlation of Mediterranean shallow water deposits (Mutti et al., 1997, 1999, 2006; Brandano et al., 2010). To assess the influence of local vs. global factors in the Oligocene–Miocene central Mediterranean Sea, we re-evaluate the biostratigraphy as well as oxygen and carbon stable isotope trends from the Decontra section and complement the multi-proxy data set with CaCO_3_, total organic carbon, magnetic susceptibility and gamma-ray data.

## 2 Geological setting and Oligocene–Miocene stratigraphy

**Fig. 1:**
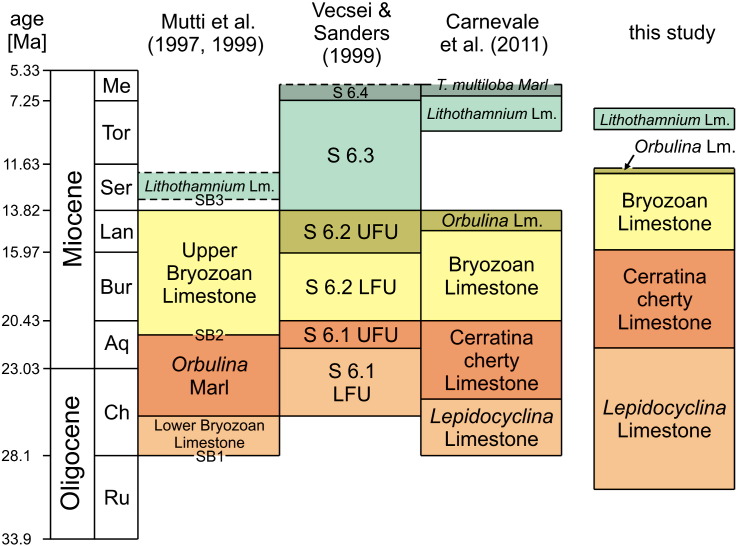
Comparison of the various informal lithostratigraphic units and present age models for the Oligocene–Miocene Bolognano Formation on the Maiella carbonate platform; chronostratigraphy according to Gradstein et al. (2012), SB = boundaries of depositional sequences (Mutti et al., 1999).

The Maiella Platform is a long-lived carbonate platform (Jurassic–Miocene) at the northern edge of the isolated Apulia Platform. The last phase of carbonate deposition is represented by the up to 200-m-thick Bolognano Formation (Oligocene–Miocene), which was deposited on a low inclined ramp at the northwestern platform margin (Vecsei et al., 1997; Mutti et al., 1999). The Bolognano Formation is subdivided into various informal members (Crescenti et al., 1969; Mutti et al., 1999; Vecsei and Sanders, 1999; Carnevale et al., 2011; Fig. 1). According to Mutti et al. (1997, 1999) three depositional sequences, including shallow water to deeper water sediments, were differentiated in the northwestern Maiella. The first depositional sequence is unconformably overlying Eocene limestones (Vecsei and Sanders, 1999). It starts with cross-bedded, bioclastic grainstones and rudstones of the Lower Bryozoan Limestone. Due to the dominance of lepidocyclinids this informal lithostratigraphic unit is also named *Lepidocyclina* Limestone by several authors (Merola, 2007; Carnevale et al., 2011; Brandano et al., 2012). It is overlain by siliceous hemipelagic marls and marly limestones (*Orbulina* Marls sensu Mutti et al., 1997, 1999; Cerratina cherty Limestone of Carnevale et al., 2011). The second depositional sequence begins with a monotonous succession of cross-bedded grainstones dominated by planktonic foraminifers, bryozoans, and echinoderms (Upper Bryozoan Limestone of Mutti et al., 1997, 1999) and ends with *Orbulina*-rich marls (*Orbulina* Marls sensu Mutti et al., 1997, 1999; *Orbulina* Limestone of Carnevale et al., 2011). The hemipelagic deposits in the upper part of the first and second depositional sequences (*Orbulina* Marls/Cerratina cherty Limestone, *Orbulina* Limestone) are wedge-shaped in cross-section and disappear toward the platform in the SE (Mutti et al., 1999). Due to its lesser SE-extent, the second hemipelagic interval does not occur in the section studied by Mutti et al. (1997, 1999; Decontra section) (Fig. 1). The second depositional sequence is unconformably overlain by the *Lithothamnium* Limestone, which is composed of coralline red algae, benthic foraminifers and molluscs followed by hemipelagic marls (*Orbulina* Marls according to Mutti et al., 1997, 1999; *Turborotalia multiloba* Marl of Carnevale et al., 2011). These informal lithostratigraphic units form the third depositional sequence of Mutti et al. (1997, 1999). The uppermost interval *Orbulina* Marls/*T. multiloba* Marl is not exposed in the Decontra section (Mutti et al., 1997, 1999; this study; Fig. 1) but laterally present.

In terms of sequence stratigraphy, the Bolognano Formation is interpreted as a (2nd-order) supersequence that is subdivided into four 3rd-order sequences (S 6.1–S 6.4; Bernoulli et al., 1992; Vecsei et al., 1997; Vecsei and Sanders, 1999; Fig. 1). Each 3rd-order sequence is composed of bryozoan and corallinacean dominated skeletal limestones in its lower part, representing the TST, and planktonic foraminifera-rich marly limestones in its upper part, representing the HST (Vecsei and Sanders, 1999). The age constraints of these 3rd-order sequences are, however, insufficient for correlation with the global sea level curve (Vecsei and Sanders, 1999).

To overcome the poor biostratigraphic resolution of the Bolognano Formation, Mutti et al. (1997, 1999) established a strontium isotope chronology for the Decontra section. Following this, the Lower Bryozoan Limestone/*Lepidocyclina* Limestone started immediately before 26.5 Ma and lasted to 26.2 Ma (Chattian; Fig. 1). It also indicates a late Aquitanian to middle Langhian age for the second depositional sequence of Mutti et al. (1997, 1999; Fig. 1). Conflictingly with this interpretation, Mutti et al. (1997, 1999) document *Orbulina* Marls at the top of the first depositional sequence (Fig. 1). The first occurrence of the foraminiferal genus *Orbulina* is, however, reported from the Langhian (Wade et al., 2011). Moreover, the age of the *Lithothamnium* Limestone is ambiguous (?Serravallian), because strontium isotope ages are inconsistent (Mutti et al., 1999). Contradictory to the strontium chronology the Lower Bryozoan Limestone/*Lepidocyclina* Limestone has been dated to the late Rupelian based on the biometric analyses of two *Nephrolepidina praemarginata* populations (Benedetti et al., 2010) and the *Lithothamnium* Limestone has been dated to the Tortonian p.p.–Messinian p.p. based on planktonic foraminifers (Merola, 2007; Carnevale et al., 2011; Fig. 1).

## 3 Locality and methods

**Fig. 2:**
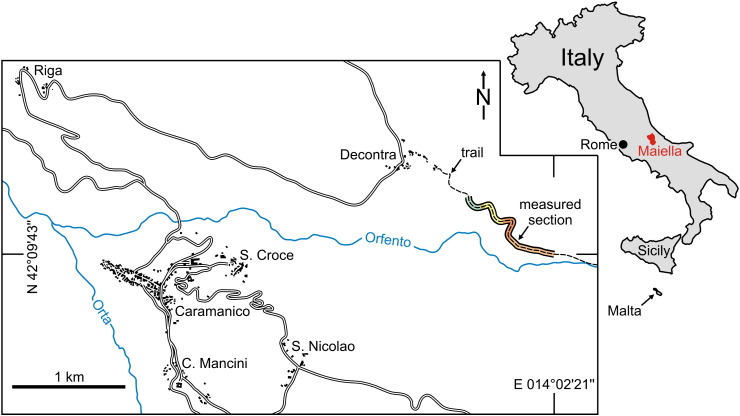
Location map showing the position of the measured section at the village Decontra (northeastern margin of the Maiella Platform, central Italy). The colours indicating the section refer to the informal lithostratigraphic units in Fig. 1.

The study site at the northwestern side of the Maiella Mts. is located 1 km southeast of the village Decontra along a trail at the northern slope of the Orfento river valley (base of the measured section: N 42°09′43.5″, E 014°02′21.6″; Fig. 2). The 120-m-thick Decontra section was measured bed-for-bed and 121 thin sections (5 × 5 cm) were prepared for microfacies analyses. Total gamma radiation and magnetic susceptibility were measured in the field with a portable “GS-512” gamma-ray spectrometer (SatisGeo; measuring time 20 s) and a handhold “SM-20” magnetic susceptibility meter (GF Instruments) with a sensitivity of 10^− 6^ SI units. The distances between the geophysical point measurements were 10 cm (GR) and 5 cm (MS). A total of 89 bulk sediment samples were measured for δ^18^O and δ^13^C at the Institute for Earth Sciences at the University of Graz, using an automated Kiel II preparation line and a Finnigan MAT Delta Plus mass spectrometer. Dried samples were powdered and reacted with 100% phosphoric acid at 70 °C. Analytical precision, based on replicate analysis of international standards NBS-19 and NBS-18 and an internal laboratory standard, is better than 0.08‰ for δ^18^O and 0.04‰ for δ^13^C. Results are reported in conventional δ-notation relative to the Vienna Pee Dee Belemnite standard (VPDB) in ‰ units. To verify extreme values we performed repeated measurements. Additionally, the sample powders were analysed for total carbon (TC) and total organic carbon contents (TOC, after acidification of samples to remove carbonate) using a Leco CS-300 analyser. The difference between TC and TOC is the total inorganic carbon content (TIC). TIC contents were used to calculate calcite equivalent percentages (= 8.34 ∗ TIC). Further geochemical proxies (Sr/Ca, Mg/Ca) have not been applied since no isolated calcareous skeletons were available.

Lithostratigraphy is used according to the informal classification of Carnevale et al. (2011; Fig. 1). Older lithostratigraphic schemes differentiated three units *Orbulina* Marls (Bernoulli et al., 1992; Mutti et al., 1997, 1999). Their use is, however, misleading, since none of them is a marl and one even does not contain *Orbulina*.

## 4 Results

### 4.1 Facies

**Fig. 3:**
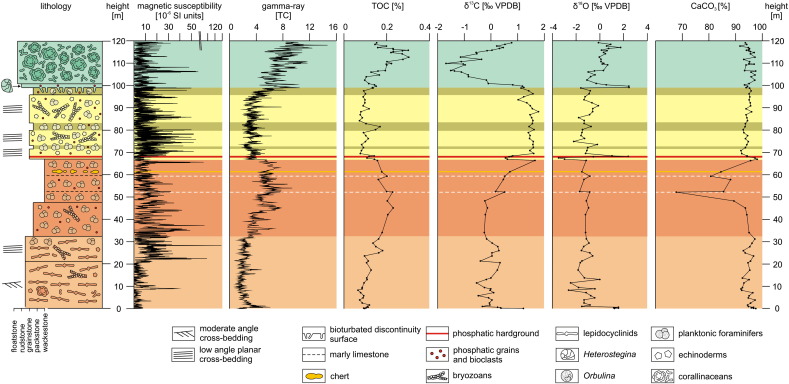
Lithological log and multi-proxy trends. The colours of the depositional units correspond to the informal lithostratigraphic units in Fig. 1.

**Fig. 4:**
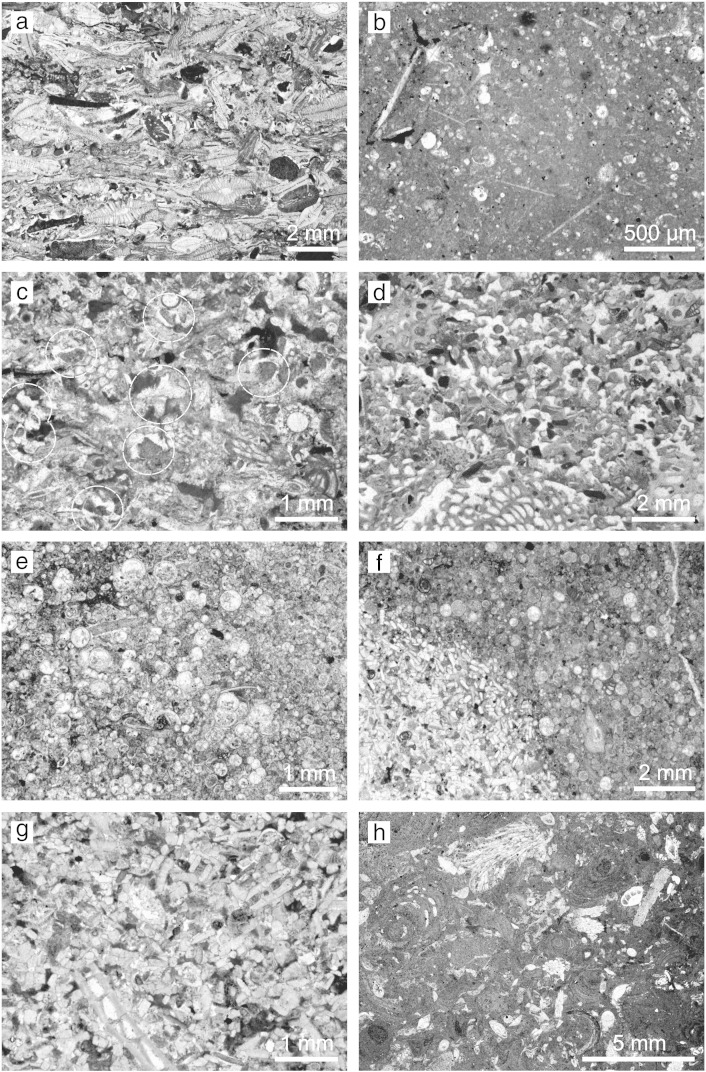
Microfacies. (a) *Lepidocyclina* rudstone with *Amphistegina*, bryozoan and echinoderm clasts; upper part of the *Lepidocyclina* Limestone. (b) Planktonic foraminifera wackestone with abundant sponge spicules; upper part of the Cerratina cherty Limestone. (c) Bryozoan-echinoderm packstone with planktonic foraminifers and common ophiurid elements (white circles); Bryozoan Limestone. (d) Microbial micrite at the phospatic hardground in the lower part of the Bryozoan Limestone. (e) Planktonic foraminiferal packstone with *Orbulina*; *Orbulina* Limestone. (f) *Heterostegina* debris as infilling of *Thallassinoides* burrows at the top of the *Orbulina* Limestone. (g) Bioclastic *Heterostegina* grainstone; base of the *Lithothamnium* Limestone. (h) Corallinacean floatstone composed of corallinacean branches and bryozoans. The bioclastic packstone matrix is rich in small benthic foraminifers and echinoid spines; *Lithothamnium* Limestone.

**Fig. 5:**
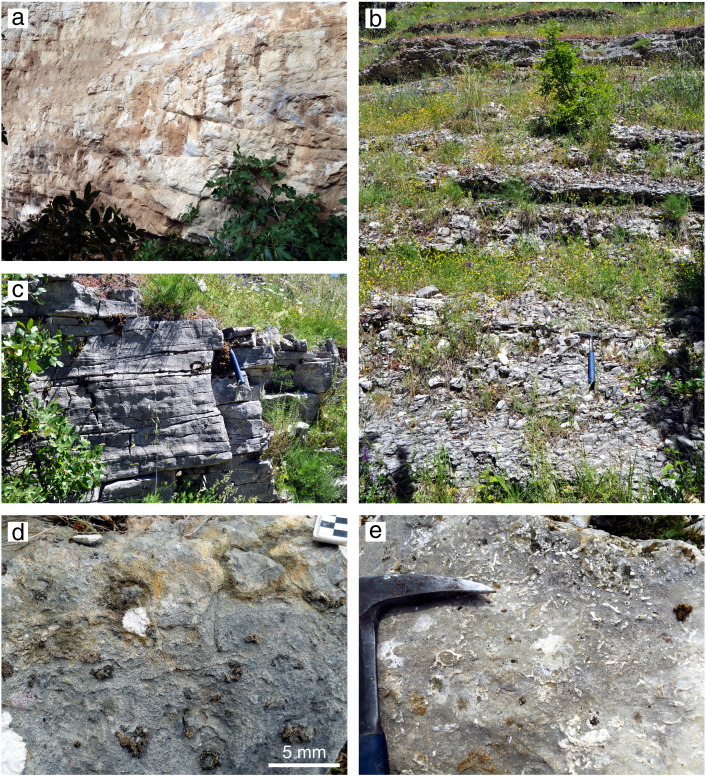
Field aspects of Decontra section. (a) Moderate angle cross-bedding in the lower part of the *Lepidocyclina* Limestone (b) Horizontally bedded planktonic foraminiferal limestones of the Cerratina cherty Limestone. (c) Low angle planar cross-bedded Bryozoan Limestone. (d) Phosphatic hardground in the lower part of the Bryozoan Limestone. (e) Corallinacean floatstone; *Lithothamnium* Limestone.

The measured section of the Bolognano Formation (Fig. 3) starts with a 32-m-thick depositional unit of bioclastic packstones, grainstones and rudstones dominated by lepidocyclinids and other larger benthic foraminifers (*Amphistegina*, nummulitids; *Lepidocyclina* Limestone; Fig. 4a) that is unconformably overlying Eocene limestones. Small benthic foraminifers and abraded bryozoan, mollusc, echinoderm and corallinacean fragments are associated. The lower part of the *Lepidocyclina* Limestone (0–21 m in the section) is characterized by moderate angle (20–30°) cross-bedding (Fig. 5a) and the occurrence of *Nephrolepidina praemarginata*. In contrast, its upper part shows low-angle (< 10°) planar cross-stratification and an upsection increasing amount of planktonic foraminifers. *Nephrolepidina morgani* replaces *N. praemarginata* in this part of the section.

Above follows a 35-m-thick succession of horizontally bedded bioturbated, fine bioclastic planktonic foraminiferal limestones with small amounts of echinoderm and bryozoan clasts, radiolarians and siliceous sponge spicules (Cerratina cherty Limestone; Figs. 3, 4b, 5b). Phosphate fillings in foraminiferal chambers are common (Mutti et al., 1997). The lower part of the Cerratina cherty Limestone (32–47 m in the section) is characterized by grain- and packstones, while its upper part consists of wackestones interbedding with thin calcareous marl and argillaceous limestone layers. Spiculitic chert nodules occur in some horizons. The first occurrence of *Praeorbulina* in Decontra section is in the uppermost part of the Cerratina cherty Limestone.

With a sharp contact the section continues with a 32-m-thick succession of low angle planar cross-bedded bioclastic grain- and packstones dominated by bryozoans, echinoderms (i.e. ophiurids) and planktonic and benthic foraminifers in variable portions (Bryozoan Limestone, 67–96 m in the section; Figs. 3, 4c, 5c). Phosphatic grains and phosphatized bioclasts are also common. The basal 2 m of the Bryozoan Limestone are characterised by a low plankton content and the abundant occurrence of *Elphidium*, *Amphistegina*, miliolids and corallinaceans. This sediment package ends with a 0.2-m-thick phosphatic hardground (Fig. 5d), which is described in detail by Mutti and Bernoulli (2003). A notable feature is the occurrence of microbial micrite in the sediment pore space directly below the hardground surface (Fig. 4d; Mutti and Bernoulli, 2003). Above the hardground, two intervals of planktonic foraminiferal limestones (packstones and grainstones) are intercalated in the Bryozoan Limestone at 72 m and from 80 to 83 m in the section.

A third 3-m-thick interval of planktonic foraminiferal pack- and grainstones with abundant *Orbulina* (*Orbulina* Limestone) occurs on top the Bryozoan Limestone (Figs. 3, 4e). It is overlain by a 1.5-m-thick bioclastic *Heterostegina* grainstone with phosphatic grains representing the base of the totally 20-m-thick *Lithothamnium* Limestone succession. The contact between the *Orbulina* Limestone and the *Lithothamnium* Limestone is a sharp surface from which *Thallassinoides* burrows intrude into the underlying sediment (Fig. 4f). This surface is considered to indicate an interruption of sedimentation (Mutti et al., 1997; Carnevale et al., 2011). The *Lithothamnium* Limestone continues thick-bedded corallinacean packstones, rudstones and floatstones (Figs. 4h, 5e). Corallinaceans are represented by branches (Fig. 5e) and rhodoliths. Larger (mostly amphisteginids, some operculinids and heterosteginids) and smaller benthic foraminifers (*Elphidium*, rotalids), molluscs, bryozoans, echinoids, serpulids (*Ditrupa*) and brachiopods are associated.

### 4.2 Geochemical and geophysical proxy trends

#### 4.2.1 CaCO_3_, magnetic susceptibility, gamma-ray and total organic carbon

A CaCO_3_ content over 90% is detected in most parts of the Decontra section. Exceptional is only the upper part of the Cerratina cherty Limestone, where it decreases to 70–90% (Fig. 3). In contrast, the section records relatively low magnetic susceptibility (MS), gamma-ray (GR) and total organic carbon (TOC) values (Fig. 3). The lowest MS occurs in the lower part of the *Lepidocyclina* Limestone. An abrupt MS increase occurs together with the change from moderate angle to low angle cross-bedded facies in the upper part of the *Lepidocyclina* Limestone (Fig. 3). Subsequently, the MS log shows a generally decreasing trend towards the top of the Cerratina cherty Limestone with distinct MS minima at the transition *Lepidocyclina* Limestone–Cerratina cherty Limestone (Fig. 3). In the above following Bryozoan Limestone to *Orbulina* Limestone succession prominent MS minima coincide with planktonic foraminiferal dominated intervals and prominent MS maxima with bryozoan and echinoderm dominated intervals (Fig. 3). The *Lithothamnium* Limestone shows a random MS pattern. GR and TOC trends are positively correlated with negative excursions in grain-supported, cross-bedded facies (*Lepidocyclina* Limestone, Bryozoan Limestone) and positive excursions in micrite-rich facies (Cerratina cherty Limestone, *Lithothamnium* Limestone; Fig. 3).

#### 4.2.2 Stable oxygen and carbon isotopes

The carbon stable isotope record (Fig. 3) starts with a negative δ^13^C shift. Subsequently, δ^13^C fluctuates in the range of − 0.5–0.3‰. After a 0.3‰ δ^13^C maximum in the upper part of the *Lepidocyclina* Limestone (25 m in the section), δ^13^C is gradually decreasing towards the top of the *Lepidocyclina* Limestone and stabilizes at around − 0.2‰ in the lower part of the Cerratina cherty Limestone. The upper part of the Cerratina cherty Limestone shows a gradual increase up to 1.6‰. The lower part of the Bryozoan Limestone (below the phosphatic hardground) correlates to a ~ 1‰-amplitude negative δ^13^C peak. Above the hardground δ^13^C values stabilized around 1.5‰. A low-amplitude negative δ^13^C excursion peaking at 1.0‰ follows in the upper part of the Bryozoan Limestone (88–99 m in the section). The *Lithothamnium* Limestone records a significant negative δ^13^C excursion peaking at − 1.6‰ at 110 m in the section.

Generally, δ^18^O values are more fluctuating in heterogeneous (*Lepidocyclina* Limestone, Bryozoan Limestone, *Lithothamnium* Limestone) than in homogenous sediments (Cerratina cherty Limestone; Fig. 3). However, the oxygen isotope record exhibits three positive outliers: (1) a pronounced one at the base of the section, (2) one at the phosphatic hardground in the lowermost part of the Bryozoan Limestone, and (3) one that corresponds to the *Heterostegina* grainstone at the base of the *Lithothamnium* Limestone (Fig. 3). A significant negative peak (~− 3.8‰) occurs directly below the hardground in the Bryozoan Limestone.

## 5 Discussion

### 5.1 Depositional environments

The Oligocene–Miocene platform carbonates of the central Apennines were deposited in a ramp environment (Vecsei and Sanders, 1999; Civitelli and Brandano, 2005; Brandano et al., 2012; Pomar et al., 2012). High-energy deposits associated with seagrass meadow sediments occur on the inner ramp (above the storm wave-base). In more basinward inner ramp positions corallinacean rud- and floatstones with scattered coral occurrences were deposited (Brandano et al., 2012; Pomar et al., 2012). However, neither corals nor seagrass indicators occur in the studied sedimentary succession at the Maiella platform margin. The middle ramp sediments on the central Apennine carbonate platforms formed in the mesophotic to oligophotic zone by corallinaceans, molluscs, and larger benthic foraminifers (Brandano et al., 2012; Pomar et al., 2012). In the studied section this depositional environment is represented by the *Lithothamnium* Limestone (Figs. 4h, 5e) and by the *Lepidocyclina* Limestone (Figs. 4a, 5a). For the latter Brandano et al. (2012) reconstructed a vast down-slope migrating dunefield where the main carbonate constituents (larger benthic foraminifers and corallinaceans) suggest an oligophotic setting. In contrast, light-dependent benthic biota were absent on the aphotic outer ramp. There, carbonate production was dominated by plankton and heterotrophic benthic organisms (bryozoans, echinoderms, bivalves; Brandano et al., 2012). Accordingly, the Bryozoan Limestone (Fig. 4d) is interpreted to have formed on the outer ramp. The high amount of planktonic biota in the cross-bedded bioclastic grainstones (Fig. 5c) suggests sediment shifting by bottom currents on the outer ramp (Stow et al., 1996; Martín-Chivelet et al., 2003). Bioturbated mudstones with abundant planktonic foraminifers, sponge spicules and radiolarians characterise the distal outer ramp of the Oligocene–Miocene carbonate platforms in the central Apennines (Brandano et al., 2012; Pomar et al., 2012). This setting is documented by the Cerratina cherty Limestone (Figs. 4b, 5b) and the *Orbulina* Limestone (Fig. 4e, f) in the Decontra section (Bernoulli et al., 1992; Mutti et al., 1999; Vecsei and Sanders, 1999) as well as by two intervals of planktonic foraminiferal limestones in the Bryozoan Limestone.

### 5.2 Paleoenvironmental significance of CaCO_3_ content, magnetic susceptibility, TOC and gamma-ray

The high CaCO_3_ content and weak MS, GR and TOC signals in the Decontra section (Fig. 3) display the isolated setting of the Maiella carbonate platform in the central Mediterranean Sea. Such isolated platform carbonates have a very low MS, not only because of relatively small terrigenous input but also due to dilution of terrigenous supply by biogenic carbonate (Ellwood et al., 2000). However, even in settings which are not directly influenced by continental runoff, eustatic-based erosion is considered as the primary cause of higher magnitude, low-frequency signatures in MS data (Ellwood et al., 2000). A potential source for the magnetic minerals is wind-blown dust from weathered rocks on the continents (Hladil et al., 2010). Glaciation has been proposed to increase dust flux into the atmosphere through increased surface winds, low surface humidity and soil moisture, and increased desertification as a result of decreasing sea level and decreasing vegetation (Bar-Or et al., 2008). In consistence, the gradual MS decline from the upper part of the *Lepidocyclina* Limestone to the top of the Cerratina cherty Limestone coincides with a relative sea level rise, which is documented by the transition from middle to distal outer ramp facies. Upsection, MS minima in the Bryozoan Limestone and *Orbulina* Limestone also always correspond to planktonic foraminifera-dominated distal outer ramp facies (Fig. 4e) while MS maxima coincide with shallower bryozoan and echinoderm-dominated proximal outer ramp facies (Figs. 3, 4c). In contrast, the abruptness of the strong MS shift in the *Lepidocyclina* Limestone (Fig. 3) argues against a primary eustatic cause. Notably, it coincides with the change from moderate angle to low angle cross-bedded facies (Fig. 3). Since the type of cross-bedding is primarily a result of water energy and density-related fractionation processes are an important control on the magnetic properties of sediments (da Silva et al., 2009; Hatfield et al., 2010) this sudden MS increase is probably rather a reflection of local hydrodynamics than of eustatic sea level change.

The TOC usually do not reflect primary organic productivity but the preservation and transport of organic matter. In particular, oxygen exposure controls carbon preservation in the sediments (Buridge, 2006). Thus, in carbonates the highest TOC concentrations occur in micrite-rich facies while well-winnowed skeletal facies show low values (Hunt, 1996). Accordingly, low TOC contents correspond to high energetic, grain-supported facies in the Decontra section (*Lepidocyclina* Limestone, Bryozoan Limestone; Figs. 4a, c, 5a, c) while high TOC concentrations occur in muddy, low-energy deposits (Cerratina cherty Limestone, *Lithothamnium* Limestone; Figs. 3, 4b, h, 5b, e). Remarkably, there is a close correlation of the TOC curve with the GR values (Fig. 3). Due to the low siliciclastic content in the Decontra section and thus low potassium and thorium concentrations, the GR spectrum must predominantly reflect the uranium content (Ehrenberg et al., 2008). This element is precipitated on organic particles or in association with reducing conditions, which developed by organic decomposition near the seafloor. Hence, low availability of organic matter lowers the potential for incorporation of uranium in marine sediments (Ehrenberg et al., 2008). In conclusion, the TOC and GR curves in the Decontra section are interpreted as reflection of water energy (and not of productivity and terrigenous sediment input).

### 5.3 Biostratigraphy

Although most facies in the Decontra section contain abundant plankton, it cannot be extracted from the hard lithified pure limestones. The biostratigraphic framework is therefore very rough and contains only a few tie points. Lepidocyclinids appeared not before the late Rupelian in the Mediterranean region (BouDagher-Fadel and Price, 2010). Thus, the presence of lepidocyclinids at the base of the section shows that the deposition of the Bolognano Formation could not have started before the late Rupelian at Decontra. For the lower part of the *Lepidocyclina* Limestone the occurrence of *Nephrolepidina praemarginata* indicates a late Rupelian to Chattian (SBZ22–lowermost SBZ23 of Cahuzac and Poignant, 1997) age. Accordingly, Benedetti et al. (2010) suggest a late Rupelian (SBZ22A of Cahuzac and Poignant, 1997) age for the *Lepidocyclina* Limestone according to the biometric analysis of two *Nephrolepidina praemarginata* populations from the northern Maiella. In contrast, a Chattian to early Burdigalian age (SBZ22B–lower SBZ25) is assigned for the top of the *Lepidocyclina* Limestone by *Nephrolepidina morgani*. It does not occur together with *N. praemarginata* in fossil assemblages. This constrains the age for the upper part of the *Lepidocyclina* Limestone to late Chattian–early Miocene (upper SBZ23–lower SBZ25).

The first occurrence of *Praeorbulina* and the absence of *Orbulina* suggest a latest Burdigalian age for the uppermost part of the Cerratina cherty Limestone. Although the first appearance datum (FAD) of *Praeorbulina* is still a matter of debate (Iaccarino et al., 2011; Turco et al., 2011b), the currently accepted FAD is 16.4 Ma (Wade et al., 2011). The FAD of *Orbulina* is 15.1 Ma (Wade et al., 2011). According to the planktonic foraminiferal stratigraphy of Merola (2007) and Carnevale et al. (2011) a late Tortonian age is considered for the *Lithothamnium* Limestone.

### 5.4 Global significance of the carbon isotope trends

**Fig. 6:**
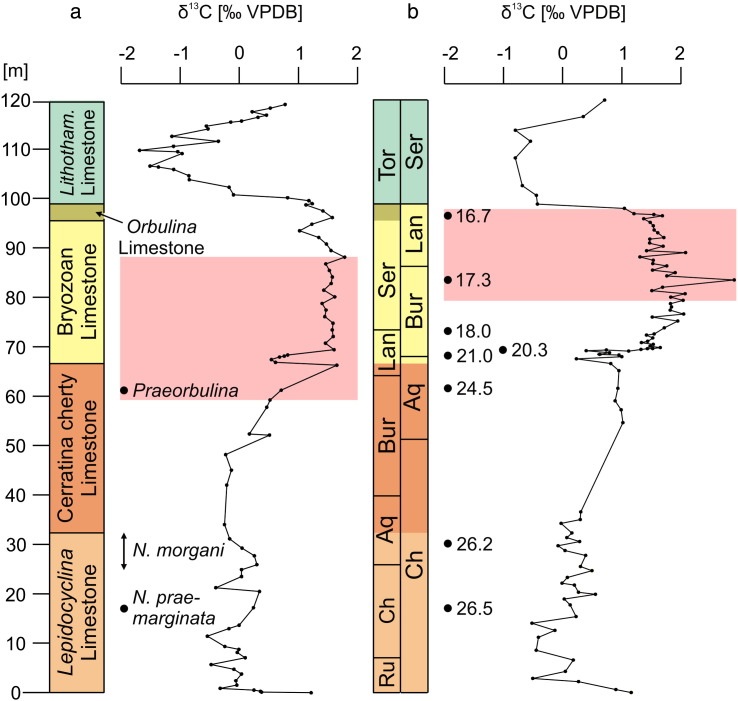
Comparison of the Decontra carbon isotope records and related age models with their stratigraphic tie points; (a) this study with biostratigraphic markers; (b) Mutti et al. (1999) with Sr-isotope ages in Ma. The Tortonian age of the *Lithothamnium* Limestone in the present study is according to the planktonic foraminiferal stratigraphy of Merola (2007) and Carnevale et al. (2011). The red bars indicate the middle Miocene global δ^13^C maximum (Monterey event).

Precipitation of carbonates involves little carbon isotopic fractionation relative to dissolved inorganic carbon (DIC) and the δ^13^C of carbonate is relatively insensitive to changes in temperature. Therefore the δ^13^C of inorganically and biologically precipitated carbonate in the oceans is very close to that of the DIC in the oceans, the largest reservoir in the recent ocean–atmosphere system (Saltzman and Thomas, 2012). The herein presented carbon isotope trends conform to those published by Mutti et al. (1997, 1999) from the Decontra section (Fig. 6), which are considered to reflect a primary marine signature. Post-depositional diagenetic alteration of the marine signal was excluded by Mutti et al. (1997) due to the absence of subaerial exposure horizons and the nonluminescent colour of skeletal carbonates under cathodoluminescence. Nonetheless, there is a significant stratigraphic offset between both curves (Fig. 6). The differences result from a mismatch between the Sr-chronology (Mutti et al., 1997, 1999) and the biostratigraphic data used in this study. The latter, however, are more reliable since they are constrained on a global scale and calibrated to the ATNTS2012 (Wade et al., 2011; Hilgen et al., 2012).

**Fig. 7:**
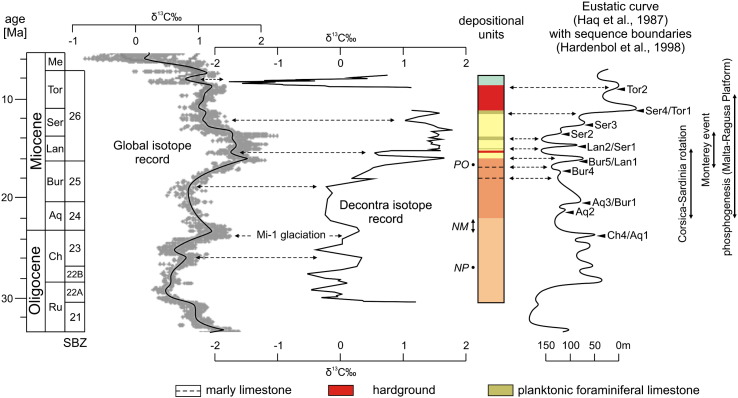
Correlation of the Oligocene–Miocene neritic carbonate record at Decontra with the global deep-sea carbon isotope curve of Zachos et al. (2001), third-order sequences of Hardenbol et al. (1998) and important paleoceanographic events; chronostratigraphy according to Gradstein et al. (2012), shallow benthic foraminiferal biozones (SBZ) of Cahuzac and Poignant (1997). The colours of depositional units refer to the informal lithostratigraphic units in Fig. 1. The occurrences of biostratigraphic markers in the measured section is indicated left to the lithostratigraphic column (*NP* = *Nephrolepidina praemarginata*, *NM* = *Nephrolepidina morgani*, *PO* = *Praeorbulina*).

Despite the increasing isolation of the Mediterranean Sea during the Miocene, there are several lines of evidence suggesting a tight coupling between the water masses of the Mediterranean Sea and the large oceanic basins during the late Oligocene to early late Miocene time interval (e.g., Jacobs et al., 1996; Kroeger et al., 2007; Föllmi et al., 2008). Accordingly, in comparing the biostratigraphically calibrated Decontra carbon isotope record with the global carbon isotope record of Zachos et al. (2001), a close correlation exists (Fig. 7). In both curves the late Rupelian is characterized by low δ^13^C values (Fig. 7). Then, both carbon isotope records show an increasing trend that is terminated by a negative peak in the late Chattian. A prominent δ^13^C maximum occurs at the Oligocene/Miocene boundary. This positive carbon isotope excursion predates the drowning of the *Lepidocyclina* Limestone ramp and coincides with the Mi-1 glaciation event in the global isotope record of Zachos et al. (2001) (Fig. 7). The subsequent gradual δ^13^C decline in the Zachos et al. (2001) carbon isotope curve, which peaks in the middle Burdigalian, is represented in the Decontra record as well. This applies also for the following gradual δ^13^C rise peaking at the Burdigalian–Langhian boundary. After this, the Langhian and lower Serravallian part of the carbon isotope records are characterized by relatively high and fluctuating δ^13^C values with a prominent negative peak in the Langhian. The amplitude of this peak is conspicuously enlarged in the Decontra section (Fig. 7). Since it correlates with the occurrence of syndepositional microbial micrite in the pore space below the phosphatic hardground in the lower part of the Bryozoan Limestone (Figs. 4d, 5d), this δ^13^C depletion can be well explained with microbial processes related to the hardground formation (Mutti and Bernoulli, 2003). A δ^13^C decline, which culminates at the Serravallian/Tortonian boundary, is indicated in both carbon isotope curves. The strong negative carbon isotope excursion in the Tortonian part of the Decontra section coincides with the significant, late Tortonian negative excursion in the global carbon isotope record. However, compared to the deep-sea record of Zachos et al. (2001) this peak is amplified in the Decontra record (Fig. 7). Because depletion of ^13^C is driven by progressive carbonate precipitation and restricted water circulation on modern shallow-water carbonate platforms (Patterson and Walter, 1994), this offset may reflect the emergence of the Maiella carbonate platform during the late Miocene. This interpretation is supported by land vertebrate fossils in the *Lithothamnium* Limestone (Scontrone, southern Maiella; Patacca et al., 2008). Importantly, extreme salinity excursions are not required for significant depletion in seawater ^13^C. Even within “near-normal” salinity range of 34‰ to 38‰ the isotopic depletion of platform seawater relative to surface open ocean water can be as great as 4‰ (Patterson and Walter, 1994).

### 5.5 Sequence stratigraphic interpretation

Considering the newly calibrated carbon isotope curve as a reference the long-term eustatic curve of Haq et al. (1988) can easily be correlated with the general trend in the Decontra section (Fig. 7). The deepening trend in the first depositional cycle of Mutti et al. (1997) (*Lepidocyclina* Limestone and Cerratina cherty Limestone) corresponds to the sea level rise from the beginning of the Chattian to the end of the Burdigalian. The highstand of this long-term eustatic cycle is indicated by the general shallowing trend that starts with the deposition of the Bryozoan Limestone at the early–middle Miocene transition and ends with the deposition of the *Lithothamnium* Limestone in the late Tortonian.

This second-order sea level change was superimposed by third-order sea level fluctuations, which are reflected in changes from planktonic to benthic carbonate production and switches from low to high MS values (Figs. 3, 7). According to the new carbon isotope stratigraphy, the transition *Lepidocyclina* Limestone–Cerratina cherty Limestone coincides with the highstand of the third-order eustatic cycle that follows on the Ch 4/Aq 1 sequence boundary of Hardenbol et al. (1998; Fig. 7). Consistently with this correlation, this part of the section exhibits a clear minimum in the MS curve (Fig. 3). The Burdigalian/Langhian boundary marks the onset of the second depositional cycle on the Maiella carbonate platform and correlates to the pronounced third-order sea level lowstand that produced the Bur 5/Lan 1 sequence boundary of Hardenbol et al. (1998; Fig. 6). Accordingly, a major relative sea level fall is documented by the massive occurrence of corallinaceans and shallow-neritic benthic foraminifers (*Elphidium*, miliolids; Leckie and Olson, 2003) at the base of the Bryozoan Limestone. The following third-order sea level highstand is documented by the first interval of planktonic foraminiferal limestones above the phosphatic hardground (Fig. 7). The second interval of planktonic foraminiferal limestone in the Bryozoan Limestone possibly corresponds to the highstand of the next third-order sea level cycle (after the Lan 2/Ser 1 sequence boundary), which was in the same magnitude (Fig. 7). The overlying *Orbulina* Limestone is constrained by the carbon isotope curve to the highstand of the third-order eustatic cycle that follows on the Ser 3 sequence boundary of Hardenbol et al. (1998; Fig. 7). The bioturbated discontinuity surface at the top of the *Orbulina* Limestone (Figs. 3, 4f) corresponds to an interval with extreme low sea level that may have interrupted carbonate production on the platform (Vecsei and Sanders, 1997). This interval started abruptly at the Ser4/Tor1 sequence boundary and continued until the Tor 2 sequence boundary in the Hardenbol et al. (1998) curve (Fig. 7). The above following corallinacean limestones correlate to the subsequent third-order sea level cycle (Fig. 7). In line with this correlation Vecsei and Sanders (1999) interpreted the *Heterostegina* debris limestone at the base of the *Lithothamnium* Limestone (Fig. 4f, g) as a transgressive lag deposit. Interestingly, recent *Heterostegina* (*Heterostegina depressa*) in the Red Sea are typical hardground dwellers that occur with the highest frequency in 40–50 m water depth (Hohenegger, 1995; Haunold et al., 1997, 1998). Accordingly, their mass occurrence at the base of the *Lithothamnium* Limestone may refer to hardground development during the non-sedimentation interval that followed after deposition of the *Orbulina* Limestone.

### 5.6 Chemostratigraphic events in the oxygen isotope record

**Fig. 8:**
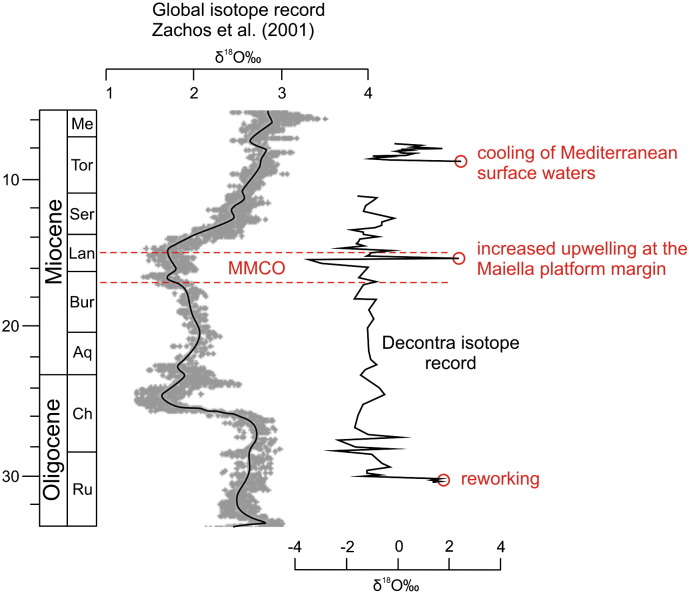
Comparison of the Decontra oxygen isotope record with the global record of Zachos et al. (2001) and correlation of significant peaks with important Oligocene–Miocene climatic events. The significant peak at ~ 9 Ma corresponds to the *Heterostegina* facies at the base of the *Lithothamnium* Limestone; MMCO = Middle Miocene Climate Optimum.

Oxygen isotope compositions are less resistant to diagenetic alteration than the carbon isotope compositions and disequilibrium fractionation in biogenic carbonate impacts oxygen isotope stratigraphy (Grossman, 2012). Accordingly, none of the global oxygen isotope events has been recognized in the Decontra oxygen isotope record so far (Mutti et al., 2006). The mainly biogenic carbonate facies contain a mixture of planktonic and benthic organisms and high amounts of skeletons that are secreted in disequilibrium with ambient seawater (e.g., larger benthic foraminifers and echinoderms). Since the corresponding oxygen and carbon isotope values do not show any correlation (r^2^ = 0.01), significant diagenetic effects are unlikely in the Decontra section. Therefore the heterogeneous composition of the carbonates may largely override the global oxygen isotope trends and events. The heavy δ^18^O values at the base of the section (Figs. 3, 8) probably reflect reworking of the Eocene and Mesozoic underground at the beginning transgression of the Bolognano Formation). In contrast, the wide range of oxygen isotope values below the phosphatic hardground at the base of the Bryozoan Limestone (Figs. 3, 8) is interpreted as effect of diagenetic processes related to hardground formation and to later diagenetic events (Mutti et al., 1997, 1999; Mutti and Bernoulli, 2003). The enrichment of ^18^O corresponds to the distribution of synsedimentary inclusion-rich calcite cements in the hardground and has been interpreted to be related to changes in the water circulation leading to intensification of upwelling at the platform margin (Mutti and Bernoulli, 2003). Notably, the new stratigraphic framework for the Decontra section places this upwelling event towards the end of the Middle Miocene Climate Optimum (~ 15–17 Ma; Zachos et al., 2001; Fig. 8), when the increasing global thermal gradient enhanced ocean circulation and upwelling (Flower and Kennett, 1994; Halfar and Mutti, 2005).

The reason for the strong positive δ^18^O peak in the *Heterostegina* facies at the base of the *Lithothamnium* Limestone (Fig. 8) is unclear. There is, however, neither petrographic nor geochemical evidence for a diagenetic overprint of the original marine isotope signature. Reworking of the underlying sediments seems also not account for this peak due to their lighter δ^18^O values (Figs. 3, 8). Increased evaporation is also unlikely to have caused enrichment of ^18^O due to the oceanic middle ramp setting and since ^18^O enriched values do not correspond to ^13^C depleted values (Patterson and Walter, 1994; Fig. 3). Therefore low water temperatures probably account for this peak. Interestingly, the carbon isotope stratigraphy constrains the age of the *Heterostegina* limestone to ~ 9 Ma. This date corresponds to an interval of temperate carbonates in the Betic intramontane basins in SE Spain (western Mediterranean Sea), which represents cooling of the Mediterranean surface waters (Braga and Aguirre, 2001; Braga et al., 2006; Fig. 8).

### 5.7 Correlation of Miocene oceanographic events in the Mediterranean Sea

The Decontra carbon isotope record is assumed as a proxy for productivity and the δ^13^C maximum in the Bryozoan Limestone was correlated with the prominent middle Miocene carbon-isotope excursion (the so-called “Monterey” event) between ~ 17 and 13.5 Ma (Mutti et al., 1999, 2006; Holbourn et al., 2007; Fig. 6B). It was first identified in the Mediterranean region in outer ramp to hemipelagic sediments on Malta (Malta-Ragusa Platform) by a major carbon excursion (amplitude of up to + 1‰) between 18 and 12.5 Ma (Jacobs et al., 1996). The old stratigraphic framework, however, predated the onset of the δ^13^C excursion by about 3 Ma in the Decontra section (Mutti et al., 1997, 1999). This time shift was interpreted as the effect of several local factors, which overlapped and partially masked the event (Mutti et al., 1999; Brandano et al., 2010). According to Mutti et al. (1997) the onset of the δ^13^C excursion in the Decontra section (Fig. 6B) reflects intensification of productivity at 21 Ma due to fertilisation of Mediterranean surface waters and increased upwelling at the platform margin in response to the collision of the Turkish and Arabian plates and platform drowning. In contrast, Brandano et al. (2010) account the Oligocene–Miocene volcanic activity in the western Mediterranean region and the closure of the Indo-Pacific marine connection as main trigger for increasing surface-water productivity in the central Mediterranean. The refined stratigraphic framework for the Decontra section, however, shifts the carbon isotope excursion towards younger ages (Figs. 6A, 7) and correlates the “Monterey” event on the Maiella and the Malta-Ragusa platforms. Despite this good correlation a limitation in time resolution remains. The 400-kyr-eccentricity modulation documented in deep water records by Holbourn et al. (2007) and simulated in the box-model of Ma et al. (2011) is clearly beyond our stratigraphic resolution. Nonetheless, the new stratigraphic framework indicates also that silica precipitation in the Cerratina cherty Limestone corresponds to a phase of huge volcanic activity in association with the Corsica-Sardinia block rotation at ~ 22 to 15 Ma (Fig. 7). This igneous activity is considered to have favoured the spreading of siliceous organisms (e.g., radiolarians, sponges) by the release of significant amounts of SiO_2_ and nutrients into the seawater, which signs responsible for siliceous sedimentation across the entire Mediterranean Sea during the early Miocene (Brandano et al., 2010). Finally, the refined stratigraphy correlates the phosphate-rich interval in Decontra section to episodes of phosphogenesis in the hemipelagic sedimentary succession of the Malta-Ragusa Platform (Fig. 6), which display relative maxima in the worldwide oceanic phosphorous burial rates at around 24 to 20, 17 to 15 and 9.5 Ma (Föllmi et al., 2008). In the Decontra section, the phosphatic hardground in the lower part of the Bryozoan Limestone corresponds to the main phase of phosphogenesis during deposition of the upper main phosphate bed on the Maltese islands at the early–middle Miocene transition (Föllmi et al., 2008).

## 6 Conclusions

In order to enable the correlation between Oligocene–Miocene shallow and deep water sediment successions in the Mediterranean region, we revised the bio- and chemostratigraphy for the 120-m-thick Decontra section at the northwestern margin of the Maiella carbonate platform in the central Apennines (Italy). The distribution of the larger benthic foraminifers *Lepidocyclina praemarginata* and *Lepidocyclina morgani* constrains a late Rupelian to early Burdigalian age for the *Lepidocyclina* Limestone. For the uppermost part of the Cerratina cherty Limestone the first occurrence of the planktonic foraminifer *Praeorbulina* and the absence of *Orbulina* indicates a latest Burdigalian age. These biostratigraphic pinning points shift the top of the *Lepidocyclina* Limestone, the Cerratina cherty Limestone and the Bryozoan Limestone towards younger ages (Fig. 1). Together with the previously reported late Tortonian age for the *Lithothamnium* Limestone (Merola, 2007; Carnevale et al., 2011), this age model reveals a strong coincidence of the shallow water carbon isotope record from Decontra with the global deep sea carbon isotope curve of Zachos et al. (2001). The multi-proxy dataset was supplemented with magnetic susceptibility (MS) and gamma-ray (GR) as well as CaCO_3_ and total organic carbon (TOC) content data. The constant high CaCO_3_ content as well as the low MS and GR values document a continuously low terrigenous discharge on the isolated carbonate platform during the studied Oligocene–Miocene time interval. GR and TOC trends correlate close. Therefore and since both proxies exhibit maxima in matrix-rich, low-energetic facies and minima in grain-supported, high-energetic facies they are interpreted as reflection of the local to regional hydrodynamic regime at the Maiella platform margin. The recorded MS trends, however, broadly correspond to facies changes reflecting relative sea level fluctuations in proximal middle to distal outer ramp settings. Based on the new carbon isotope stratigraphy, these relative sea level changes correlate well with eustatic third-order sea level cycles of Hardenbol et al. (1998). The sequence stratigraphic interpretation improved the time resolution in particular for the Bryozoan Limestone representing the middle Miocene.

According to the new integrated bio-, chemo-, and sequence stratigraphic scheme, the upwelling event, which is represented by a positive carbon isotope peak in the lower part of the Bryozoan Limestone, correlates to the end of the Middle Miocene Climate Optimum. Another significant positive oxygen isotope peak was detected at the base of the *Lithothamnium* Limestone. It possibly corresponds to a cooling episode that prevented coral reef growth in the western Mediterranean at ~ 9 Ma. The final emergence of the Maiella carbonate platform during the late Miocene is reflected by the strongly increased amplitude of the late Tortonian negative carbon isotope excursion in the Decontra record compared to the global carbon isotope curve of Zachos et al. (2001). Furthermore, the new stratigraphy allows for the first time the precise timing of important paleoceanographic events (“Monterey” event, events of phosphogenesis and volcanic SiO_2_ input) on the central Apennine carbonate platforms and their correlation with the Malta-Ragusa Platform.
